# Smooth muscle gap-junctions allow propagation of intercellular Ca^2+^ waves and vasoconstriction due to Ca^2+^ based action potentials in rat mesenteric resistance arteries

**DOI:** 10.1016/j.ceca.2018.08.001

**Published:** 2018-11

**Authors:** Lyudmyla Borysova, Kim A. Dora, Christopher J. Garland, Theodor Burdyga

**Affiliations:** a Department of Cellular and Molecular Physiology and Gastroenterology, Institute of Translational Medicine, University of Liverpool, Crown Street, Liverpool, L69 3BX, UK; b Department of Pharmacology, University of Oxford, Mansfield Road, Oxford, OX1 3QT, UK

**Keywords:** AP, action potential, SMC, smooth muscle cells, VGCC, voltage gated Ca^2+^ channels, MA, mesenteric artery, TEA, tetraethyl ammonium, 18β-GA, 18β-glycyrrhetinic acid, Intercellular Ca^2+^waves, Spreading vasoconstriction, Gap junctions, Mesenteric resistance artery

## Abstract

The role of vascular gap junctions in the conduction of intercellular Ca^2+^ and vasoconstriction along small resistance arteries is not entirely understood. Some depolarizing agents trigger conducted vasoconstriction while others only evoke a local depolarization. Here we use a novel technique to investigate the temporal and spatial relationship between intercellular Ca^2+^ signals generated by smooth muscle action potentials (APs) and vasoconstriction in mesenteric resistance arteries (MA). Pulses of exogenous KCl to depolarize the downstream end (T1) of a 3 mm long artery increased intracellular Ca^2+^ associated with vasoconstriction. The spatial spread and amplitude of both depended on the duration of the pulse, with only a restricted non-conducting vasoconstriction to a 1 s pulse. While blocking smooth muscle cell (SMC) K^+^ channels with TEA and activating L-type voltage-gated Ca^2+^ channels (VGCCs) with BayK 8644 spread was dramatically facilitated, so the 1 s pulse evoked intercellular Ca^2+^ waves and vasoconstriction that spread along an entire artery segment 3000 μm long. Ca^2+^ waves spread as nifedipine-sensitive Ca^2+^ spikes due to SMC action potentials, and evoked vasoconstriction. Both intercellular Ca^2+^ and vasoconstriction spread at circa 3 mm s^−1^ and were independent of the endothelium. The spread but not the generation of Ca^2+^ spikes was reversibly blocked by the gap junction inhibitor 18β-GA. Thus, smooth muscle gap junctions enable depolarization to spread along resistance arteries, and once regenerative Ca^2+^-based APs occur, spread along the entire length of an artery followed by widespread vasoconstriction.

## Highlights

• In rat mesenteric resistance arteries (MA), smooth muscle (SMC) gap junctions enable intercellular Ca^2+^ waves and vasoconstriction.
• Simultaneous block of K^+^ channels and activation of L-type VGCCs triggered SMC action potentials and propagating intercellular Ca^2+^ waves.
• Ca^2+^ spread was spike-like in appearance, of constant speed at 2.6 ± 0.3 mm s^−1^ and associated with vasoconstriction spread at 2.5 ± 0.3 mm s^−1^.
• The ability of arteries to spread intercellular Ca^2+^ waves and phasic contractions was independent of the endothelium.
• Propagation but not the generation of Ca^2+^ spikes was reversibly inhibited by the gap junction blocker 18β-glycyrrhetinic acid (18β–GA).

## Graphical abstract

**Figure:**
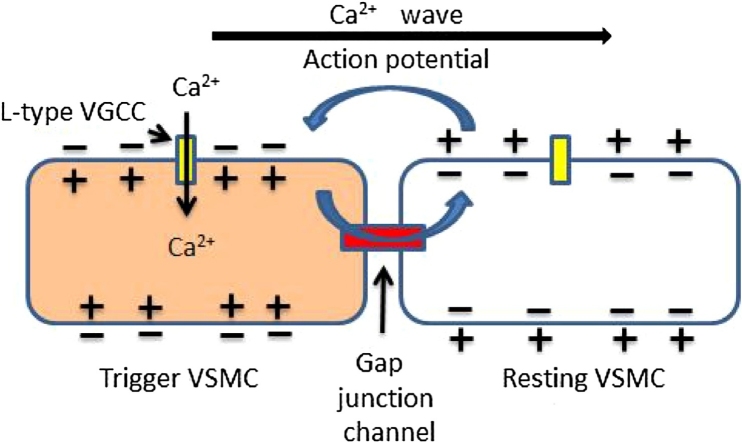

## 1 Introduction

Cell-to-cell coupling via gap junctions provides a mechanistic basis for electrical coupling between vascular cells, such that depolarizing and hyperpolarizing electrical signals are able to spread along the vessel wall to coordinate myogenic responses [[1], [2], [3], [4], [5], [6], [7]]. However, the extent of current spread appears to vary depending on the cell type stimulated, so while the endothelium conducts a change in membrane potential over considerable distance, changes in smooth muscle membrane potential appear very restricted, at least in the small arteries of skeletal muscle [[8], [9], [10]]. However, as the endothelium and SMCs are coupled via myoendothelial gap junctions, a change of potential in one cell type can pass to the other. As a consequence, although direct SMC depolarization to KCl seems poorly conducted, spread to the endothelium enables extensive intercellular conduction [11]. The SMCs are coupled effectively, as current spread can be measured in endothelium-denuded arteries [12] and arterioles [6]. However, the circumferential orientation of the cells and high intercellular resistance and current dissipation across the cell membrane is suggested to explain the relatively rapid decline in electrical signal [13].

In the presence of TEA (5–10 mM), arterial SMCs have been shown to generate spike-like APs [[14], [15], [16], [17], [18]], sensitive to L-type VGCC blockers [17,18]. However, it was not clear whether these Ca^2+^ -mediated APs can propagate via gap junctions to generate regenerative intercellular Ca^2+^ waves similar to those observed in visceral smooth muscles [19,20]. Block of SMC K^+^ channels would reduce current dissipation across the cell membrane, and as such be predicted to enhance intercellular spread of current [7]. Arterial gap junctions in both resistance and conduit arteries express Cx 40, Cx37, Cx43, and to a lesser extent Cx45 [[21], [22], [23], [24], [25], [26], [27], [28], [29], [30], [31]]. Cx40 is focussed in the endothelium, providing tight electrical coupling between these cells [[32], [33], [34]], and with SMCs through myoendothelial gap junctions [22,25,33,35,36]. How these connexins influence intercellular communication through gap junctions and as such determine the ability of arteries to propagate vasoconstriction remains incomplete. In the present study, we investigated the temporal and spatial relationship between propagating intercellular Ca^2+^ waves mediated by SMC action potentials (APs) and the ensuing vasoconstriction. APs were triggered by direct SMC depolarization to local application of KCl in the presence of TEA and BayK 8644. We used a novel technique to allow the simultaneous measurement of Ca^2+^ and force at two ends of long isolated segments of resistance artery, showing that gap-junctions can allow the free movement of propagating vasoconstriction due to AP-mediated Ca^2+^ influx.

## 2 Material and methods

### 2.1 Ethical approval

Ethical approval was obtained and all procedures were carried out in accordance with the UK Home Office Animals (Scientific Procedures) Act 1986. Experiments were performed according to the guidelines outlined by the institution’s animal welfare committee and regulations described in the Journal of Physiology editorial [37].

### 2.2 Tissue preparation

Wistar rats 225–250 g of either sex were humanely killed in accordance with UK legislation as specified by Schedule 1 of the Animals (Scientific Procedures) Act by increasing concentration of CO_2_ followed by cervical dislocation as a confirmation of death.

The mesenteric arcade was placed in HEPES buffered solution containing the following (in mM): 120.4 NaCl, 5.9 KCl, 1.2 MgSO_4_, 2 CaCl_2_, 10 HEPES and 10 glucose, pH adjusted to 7.40 ± 0.02 with NaOH (at 23 °C). Sections of mesenteric arterial arcades were carefully dissected and cleaned of adherent tissue.

### 2.3 Calcium and force measurements

Mesenteric arcades were loaded with 15 μM fluo-4 AM (Invitrogen) and 0.01% pluronic F-127 (Sigma–Aldrich) in HEPES buffered solution for 90 min at room temperature, then left to de-esterify for 30 min. After de-esterification, the mesenteric arcade was pinned in a Petri dish and segments of arteries (∼3.5 mm long, i.d.150–300 μm) were dissected. Artery segments were pulled over a 50 μm diameter stainless steel rod and carefully fixed to the bottom of a custom-made small experimental chamber (0.5 ml) using aluminium foil clips glued to the bottom of the chamber. We used a fast Nipkow disc-based confocal imaging system attached to a high sensitivity (iXon Andor) CCD camera, which allowed acquisition of images at 33–70 fps and thereby accurate measurement of temporal and spatial characteristics of Ca^2+^ signalling in mesenteric arcades. Data acquisition was performed using Andor iQ software. Images were acquired using an inverted microscope, equipped with air immersion x2 (N.A.0.04), x4 (N.A. 0.08), x10 (N.A.0.4), x20 (N.A. 0.72) and water immersion x40 (N.A.1.2) objectives (Olympus, UK).

**Fig. 1:**
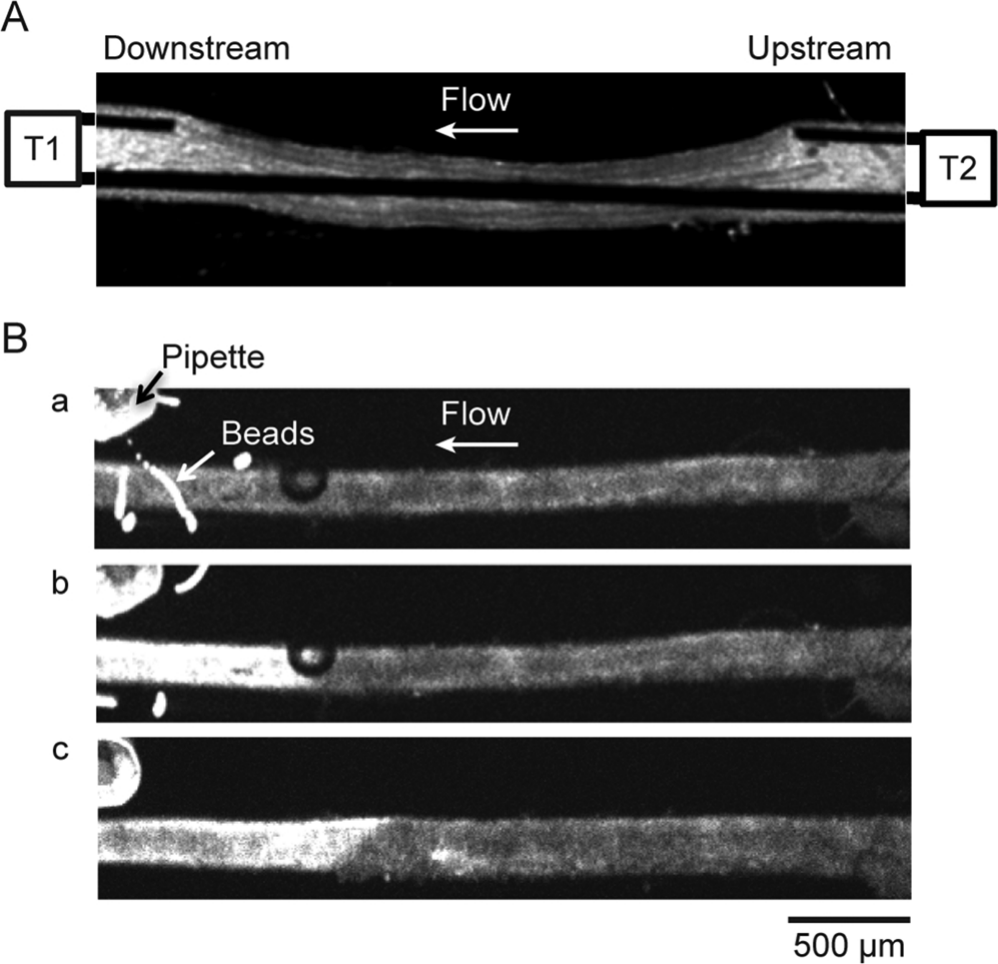
Experimental set up for simultaneous measurements of smooth muscle intercellular Ca^2+^ and force at the downstream (T1) and upstream (T2) ends of the rat mesenteric artery. ***A***, Transmitted light image showing the position of the wires used to radially stretch and measure force at T1 and T2. ***B***, Confocal fluorescence images of an artery loaded with Ca^2+^ indicator showing a local application via delivery pipette to T1 of fluorescently labelled beads (***a***), 60 mM KCl (***b***), and 5 mM caffeine (***c***). The contraction of the downstream end of the artery (T1) seen as local deflection of the wires evoked by 10 s 60 mM KCl pulse described in *A* can be seen in *Supplement Movie 1*. Spatial spread of fluorescently labelled beads described in *Ba* and Ca^2+^ signal induced by 10 s 60 mM KCl pulse described in *Bb* can be seen in *Supplement Movie 2*.

Isometric force was independently measured at downstream (T1) and upstream (T2) ends of the mounted artery (Fig. 1*A*, Supplement Movie 1) using highly sensitive force transducers (FORT 10, WPI) attached to 3D manual manipulators (U-3C, Narishige, Japan). A short section of 50 μm steel wire bent at 90° was attached to the force transducer via a stainless-steel lever extension and was carefully inserted into the lumen of arteries, positioned at each end (T1, T2, Fig. 1*A*, Supplement Movie 1). The overall length of the artery used for tension measurements was about 500 μm at each end, set to a resting tension of 0.5 mN mm^−1^ at T1 and T2. The arteries were continuously superfused with HEPES buffered solution at 2–3 ml min^−1^.

To examine a possible role of endothelium in KCl-induced propagating Ca^2+^ waves, endothelial cells were damaged by gentle rubbing of the luminal surface of artery segments with a 50 μm stainless steel wire, before they were mounted on the force transducers. Using fine tweezers an artery segment was gently rotated around thin wire for a minute. After taking the arterial segment off the wire the damaged end held by tweezers was cut off and the remaining ∼3 mm long artery segment was mounted. Two tests were performed to ensure a selective damage of the endothelium but not the SMC layer. The integrity of the endothelial layer was tested using carbachol (CCh, 1 μM) added to MAs preconstricted with 1 μM phenylephrine. The failure of CCh to terminate phenylephrine-induced Ca^2+^ oscillations and force confirmed the endothelium damage. SMCs exhibited phenylephrine-induced Ca^2+^ oscillations associated with constriction, terminated by NO donor SNAP, indicative of the SMCs viability.

### 2.4 Focal delivery of agents

Two 8 channel Pressurized Perfusion Systems (Digitimer, USA) with eight-into-one micro-manifold, combining 8 tubes into a single, removable 100 μm delivery tip were used for local application of vasoconstrictors at a pressure of 3.5 psi. Each delivery tip was held with a manual 3D micromanipulator (U-3C, Narishige, Japan) and positioned perpendicularly to the vessel axis at 5–10 μm from the vessel wall. The downstream delivery tip was positioned near the force transducer wires at T1 aside from T2 at least by 3 mm (Fig. 1*A*).

*Fluorescent beads*. In the preliminary experiments designed to define the spatial spread of the ejected solution and flow direction, 10 μm fluorescently labelled beads (FluoSperespolysterine, Life Technologies, USA) were added to 60 mM KCl. The beads were ejected from the delivery tip at downstream end of the artery (T1) against the flow at 2-3 ml min^−1^ and indicated KCl-induced Ca^2+^ transient spread upstream up to 500 μm from the delivery tip (Fig. 1*Ba* and *b*, Supplement Movie 2). In addition, another contractile agent caffeine was locally applied at T1, which is less likely to evoke depolarization and conducted constriction in contrast to KCl (Fig. 1B*c*). Local stimulation with 5 mM caffeine was repeated at least two times and indicated caffeine-induced Ca^2+^ transient spread upstream up to 500 μm from the delivery tip (Fig. 1B*c*).

**Fig. 2:**
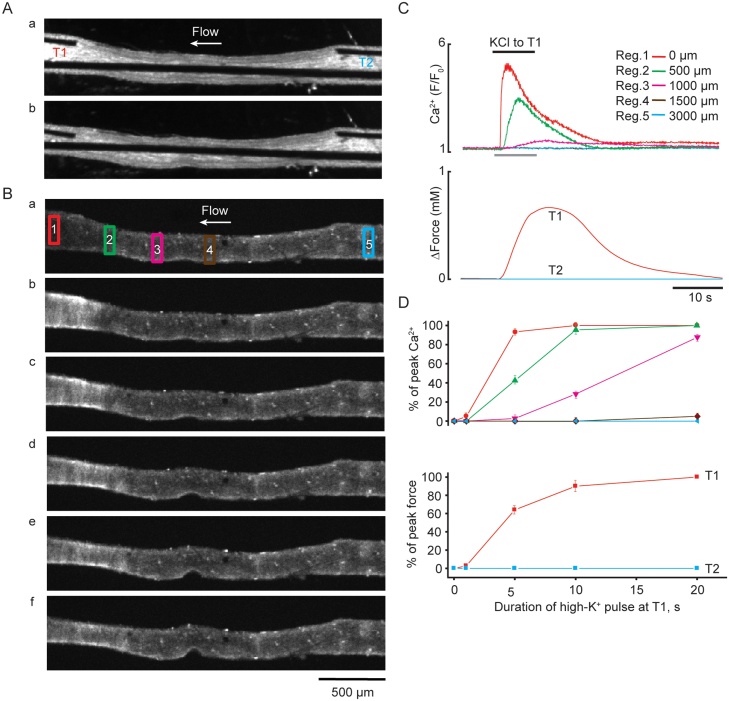
Ca^2+^ signalling and vasoconstriction induced by 60 mM KCl pulses of different duration applied at T1. ***A***, Transmitted light images of the artery (***a***) at rest and during (***b***) local stimulation at T1 with 60 mM KCl 10 s pulse. Note the deflection of the wires. ***B***, Fluorescence images of an artery loaded with Ca^2+^ indicator, recorded at rest (***a***) and during local KCl 10 s application at T1 (***b-f***). Time interval between images is 2 s. ***C***, Superimposed traces of KCl induced Ca^2+^ signal (top panel) measured in five ROIs shown in B*a* and force (bottom panel) recorded at T1 (red trace) and T2 (blue trace) (*n=*5-7). ***D***, Graph showing time-dependent effects of the local KCl application (1 s, 5 s, 10 s and 20 s duration) at T1 on the amplitude and spatial spread of Ca^2+^ signal (top panel) measured in five ROIs shown in *Ba* and force (bottom panel) recorded at T1 (red trace) and T2 (blue trace) (*n=*5–7). The spatial spread of the Ca^2+^ signal induced by 60 mM KCl 10 s pulse can be seen in *Supplement Movie 3* (For interpretation of the references to colour in the text, the reader is referred to the web version of this article).

*60 mM KCl pulse*. In each experiment, local application of 60 mM KCl for 1–20 s to the downstream end of arteries (T1) was repeated at least three times to ensure (1) Ca^2+^ signal and contractile responses were recorded solely at T1, not reaching upstream end of the artery (T2), and (2) reproducibility of the results (Fig. 2, Supplement Movies 1 and 3). All experiments were performed at 23^º^C and 37^º^C. The speed of propagation of the Ca^2+^ waves was slightly affected by a higher temperature. However, due to significant leakage of fluo-4 from SMCs at 37 °C the data presented were obtained at 23^º^C. TEA and/or BayK 8644 were added to the bath superfusion solution.

### 2.5 Measurement of smooth muscle membrane potential

Mesenteric arcades were placed in Krebs solution containing (in mM): 118 NaCl, 25 NaHCO_3_, 3.6 KCl, 1.2 MgSO_4_ ·7H_2_O, 1.2 KH_2_PO_4_, 2.5 CaCl_2_, 11 glucose and gassed with 21% O_2_, 5% CO_2_, balance N_2_ at 37 °C. A third-order mesenteric artery (external diameter between 200 and 300 μm at 70 mmHg) was dissected free of adherent tissue and a small segment ∼2 mm long removed and mounted in a Mulvany-Halpern wire myograph (model 400 A; Danish Myo Technology, Denmark). The solution temperature was raised to 37 °C, and the artery normalized to a resting tension equivalent to that generated at 90% of the diameter of the vessel at 70 mmHg. Artery reactivity was assessed by preconstriction to phenylephrine (0.5–3 μM) followed by endothelium-dependent relaxation to acetylcholine (0.1 and 1 μM). Only vessels that relaxed by >95% were used further. The vascular smooth muscle membrane potential was measured using sharp glass microelectrodes backfilled with 2 M KCl (tip resistances circa 100 MΩ), as previously described [38]. Smooth muscle membrane potential was recorded through a preamplifier (Neurolog System; Digitimer, Ltd, United Kingdom) linked to a MacLab data acquisition system (AD Instruments Model 4e, usually at 100 Hz). All drugs were added directly to the bath.

### 2.6 Solutions

HEPES buffered solution of the following composition was used (mM): 120.4 NaCl, 5.9 KCl, 1.2 MgSO_4_, 2 CaCl_2_, 10 HEPES, 10 glucose, pH adjusted to 7.40 ± 0.02 with NaOH (at 23 °C). Krebs solution containing the following (in mM): 118 NaCl, 25 NaHCO_3_, 3.6 KCl, 1.2 MgSO_4_·7H_2_O, 1.2 KH_2_PO_4_, 2.5 CaCl_2_, 11 glucose, gassed with 21% O_2_, 5% CO_2_, balance N_2_ at 37 ^º^C. High-K^+^ solution was prepared by equivalent isosmotic replacement of NaCl by KCl. Caffeine, TEA, BayK 8644 were from Sigma. Fluo-4 acetoxymethyl ester was from Molecular Probes, Life Technologies, UK. BayK 8644 was dissolved in ethanol; maximum concentration of ethanol used when applying BayK 8644 was 0.1%. Caffeine and TEA were dissolved in the HEPES buffered solution.

### 2.7 Statistics

Results are summarized as means ± s.e.m. of *n* replicates from a different animal. Data were compared using Students’ *t* test. *P <*0.05 was considered statistically significant. Average values of Ca^2+^ wave amplitude and force, measured at downstream end of the artery with T1 transducer (T1, 0 μm) and at upstream artery end with T2 transducer (T2, 3000 μm), were expressed as a percentage of peak Ca^2+^ transient and force induced by bath application of 60 mM KCl taken for 100%.

## 3 Results

### 3.1 Ca^2+^ signalling and vasoconstriction induced by pulses of KCl

60 mM KCl was ejected onto isolated MAs from at the downstream delivery point, T1, in pulses of variable duration; 1 s, 5 s, 10 s and 20 s. Ensuing changes in Ca^2+^ signalling and vasoconstriction in arterial segments were assessed with the peak in Ca^2+^ amplitude and vasoconstriction defined as 100%. To quantify spatial spread, Ca^2+^ signal was measured in five ROIs at 0 μm, 500 μm, 1000 μm, 1500 μm, and 3000 μm along the artery (Fig. 2*Ba*).

***1 s pulse of KCl:*** was followed by a small rise in SMC [Ca]_i_ in the vicinity of the application area (∼200 μm) of 4.9 ± 0.4% of peak Ca^2+^ (*n=*5, Fig. 2D, top panel, red line). No change in [Ca^2+^]_i_ was detected at 500 μm from T1 (Fig. 2*D*, top panel, green line). The small, localized rise in intracellular Ca^2+^ was associated with vasoconstriction of 2.7 ± 0.2% (*n=*7, Fig. 2*D*, bottom panel, red line).

***5 s pulse of KCl*:** increased SMC Ca^2+^ signal by 93.4 ± 3.4% at 0 μm (*n=*5) and significant increases were now evident at 500 μm and 1000 μm upstream of the T1, with average increases of 44.4 ± 5.2%, 2.7 ± 0.1%, but with no detectable signal by 1500 μm, respectively (*n=*5, Fig. 2D, top trace). 5 s pulses of KCl pulse evoked vasoconstriction of 64.2 ± 4.7% (Fig. 2D, bottom panel, red trace).

***10 s pulse of KCl*:** further increased the amplitude and spread of Ca^2+^ (Fig. 2B*b-f,* C, D, Supplement Movie 3), with a maximal (100%) Ca^2+^ signal at 0 μm (Fig. 2C, D top panel, red line) declining to 95.4 ± 6.1%, 28.3 ± 3.9% and 0% at 500 μm, 1000 μm and 1500 μm upstream from T1, respectively (*n=*5, Fig. 2D, top panel, green, magenta and dark red lines). Vasoconstriction was also increased, to 90.1 ± 6.9% (*n=*5, Fig. 2C, D, bottom panels, red trace and line). The velocity of Ca^2+^spread was 158 ± 9 μm s^−1^ (*n=*5).

***20 s pulse of KCl*:** was followed by detectable increases in Ca^2+^ at 1500 μm distance from T1. At both 0 μm and 500 μm there was a maximal (100%) increase in Ca^2+^ (Fig. 2D, top panel, red and green lines) declining to 87.8 ± 4.5% and 4.9 ± 2.1% at 1000 μm and 1500 μm, respectively (*n=*5, Fig. 2D, top panel, magenta and dark red lines). Vasoconstriction at T1 was maximal (Fig. 2D, bottom panels, red line). Neither the Ca^2+^ signal nor vasoconstriction to KCl pulses at T1 spread beyond 1500 μm, explaining zero values in both cases at the upstream (T2) end of the artery (Fig. 2D, top and bottom panels, blue lines).

### 3.2 SMC membrane potential changes with K^+^ channel block and activation of VGCCs with TEA and BayK 8644, respectively

**Fig. 3:**
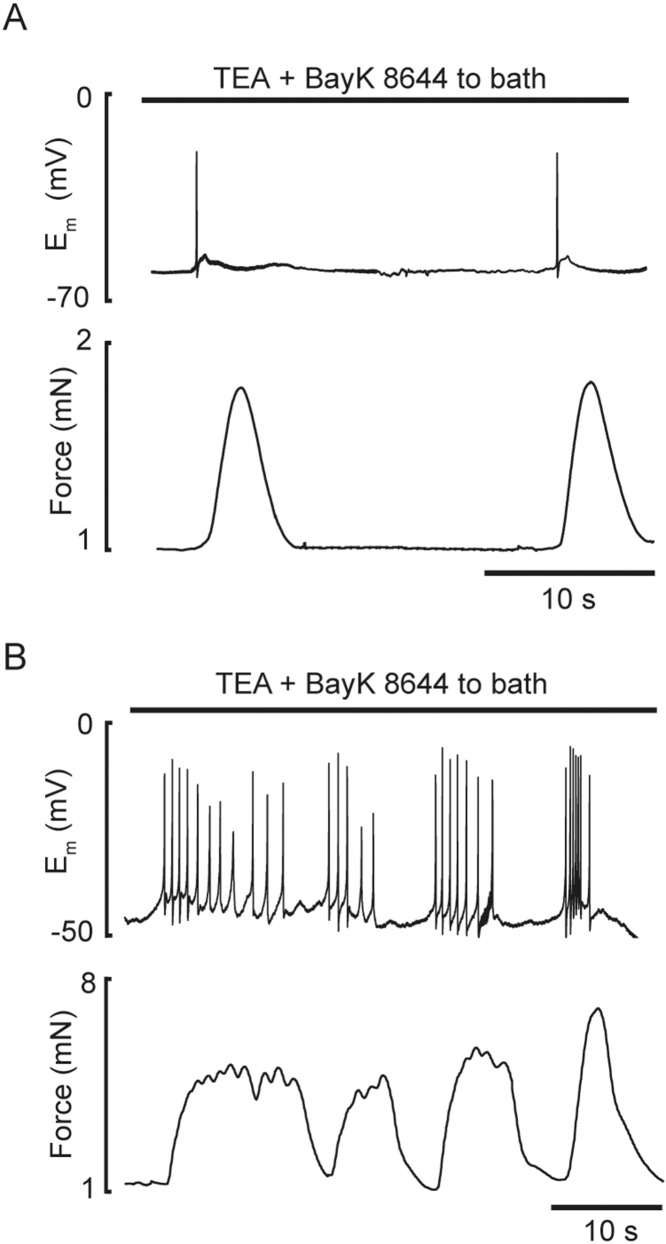
Profiles of membrane potential and tension during TEA and BayK 8644 application. Patterns of spike action potentials (top traces) and phasic contractions (bottom traces) during exposure of mesenteric artery to 10 mM TEA and 1 μM BayK 8644 (indicated by bar, *n=*4). ***A*** and ***B*** (top panel) – action potentials appearing as isolated (single) spike or bursts of spikes, respectively; ***A*** and ***B*** (bottom panel) – phasic contraction associated with single spike or burst of spikes, respectively.

Mesenteric SMCs were electrically quiescent with a membrane potential around −55 mV. Following the addition of 10 mM TEA and 1 μM BayK 8644 the membrane potential decreased to *circa* −42 mV when the SMCs became electrically active, with fluctuations in membrane potential developing into spike-like APs leading to vasoconstriction (Fig. 3A). Initially, these were solitary events, but developed into bursts of APs accompanied by group of phasic contractions, which summated producing tetanic-like contractile responses (*n=*4, Fig. 3B). Each AP had a fast upstroke followed by rapid repolarization and transient after-hyperpolarization.

### 3.3 Influence of TEA and BayK 8644 on intercellular Ca^2+^ waves in mesenteric artery arcades with intact endothelium

**Fig. 4:**
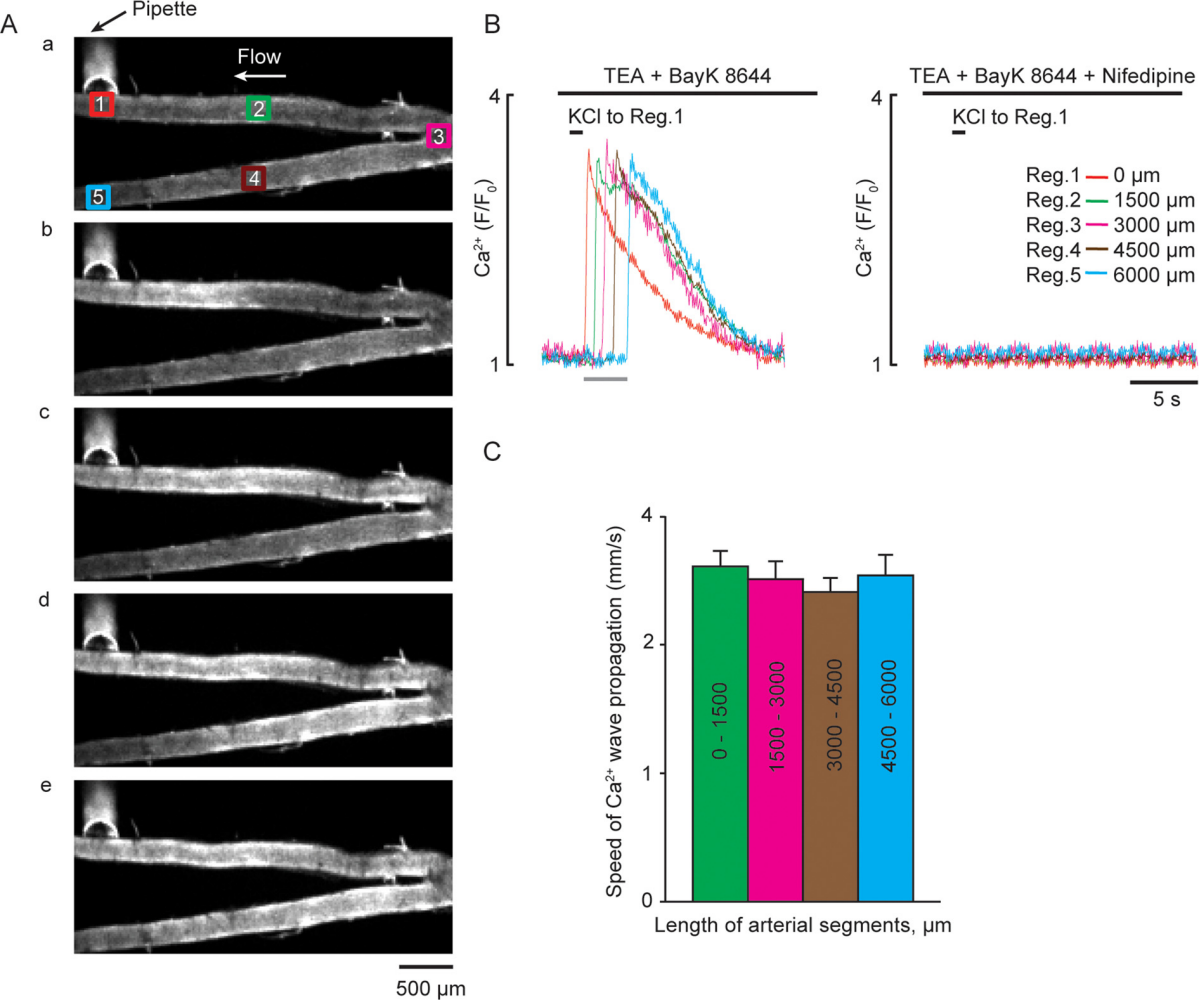
Propagating intercellular Ca^2+^ wave in the mesenteric artery arcade with intact endothelial layer, evoked by 1 s application of 60 mM KCl in the presence of TEA and BayK 8644. ***A***, Fluorescence images of arterial arcade loaded with Ca^2+^ indicator, recorded at rest (***a***) and during local stimulation with KCl applied at T1 of the 3rd order branch (***b-e***). The interval between displayed images A(a–e) is 600 ms. ***B***, Traces corresponding to Ca^2+^ signals measured in five ROIs in *Aa* spaced at 1500 μm interval recorded in the absence (*n=*3, left panel) and the presence of 10 μM nifedipine (*n=*3, right panel), respectively. The period of acquisition is indicated by grey bar. ***C***, Average speed of KCl-evoked Ca^2+^ wave propagation measured between 0–1500 μm, 1500–3000 μm, 3000–4500 μm, and 4500–6000 μm (*n=*3). The propagating Ca^2+^ wave described in A–C can be seen in *Supplement Movie 4.* (For interpretation of the references to colour in the text, the reader is referred to the web version of this article).

As SMCs of MA are electrically coupled, inhibition of K^+^ channels with 10 mM TEA and activation of L-type VGCC Ca^2+^ influx with 1 μM BayK 8644 would be likely to facilitate the generation and propagation of intercellular Ca^2+^ waves and the associated contraction. In endothelium-intact arterial arcades (second order feed branch and two branches) the presence of TEA and BayK 8644 allowed axial propagation of intercellular Ca^2+^ waves evoked by localized 1 s KCl pulses at the distal end of the arcade (T1) (*n=*3, Fig. 4A*a*). Ca^2+^ change was measured at 5 ROIs in 1500 μm increments (Fig. 4A*a*). T1 stimulation in the presence of 1 μM BayK 8644 only produced a transient local Ca^2+^ signal (Supplement, Movie 4), however in the additional presence of 10 mM TEA a regenerative Ca^2+^ intercellular wave propagated along the entire length of the arcade as a Ca^2+^ spike(s) (Fig. 4B, Movie 4 in Supporting information). The average speed of propagation was constant, 2.6 ± 0.1 (0–1500 μm), 2.5 ± 0.1, (1500–3000 μm) 2.4 ± 0.1 (3000–4500 μm) and 2.5 ± 0.2 (4500–6000 μm) mm s^−1^, respectively (*n=*3). The L-type VGCC blocker nifedipine (10 μM) fully blocked these propagating intercellular Ca^2+^ waves (*n=*3, Fig. 4C right panel).

### 3.4 Influence of TEA and BayK 8644 on intercellular Ca^2+^ waves and vasoconstriction in denuded mesenteric arteries

**Fig. 5:**
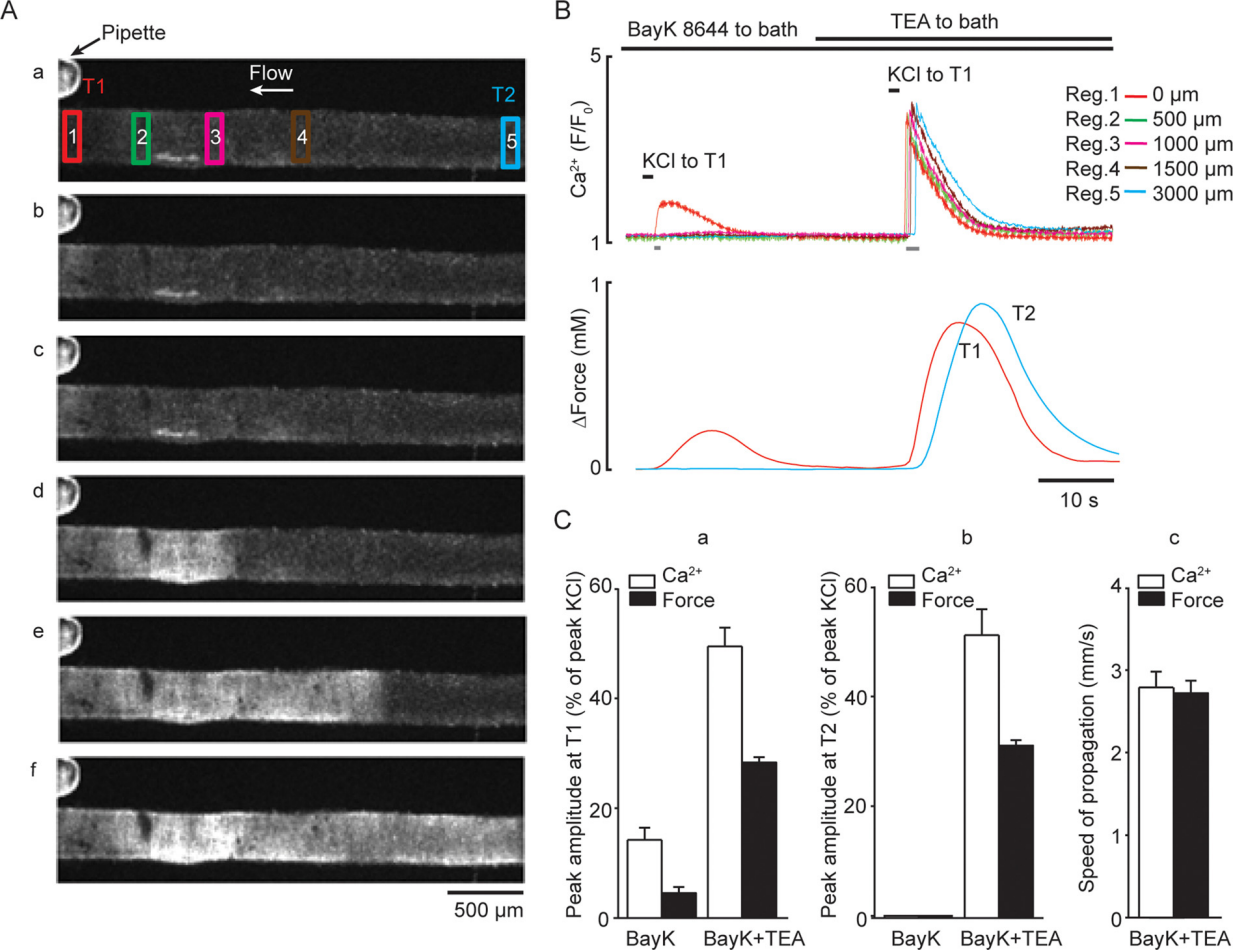
BayK 8644 alone is not sufficient to enable KCl-mediated propagating responses in denuded arteries. ***A***, Fluorescence images of an artery loaded with Ca^2+^ indicator and treated with 1 μM BayK 8644, recorded at rest (***a***) and during 1 s application of 60 mM KCl at T1 in the absence (***b***) and the presence (**c-f**) of 10 mM TEA added to the bath with 1 μM BayK 8644 (indicated by bar at *B*). The interval between displayed images A(c–f) is 700 ms. Note rapid propagation of KCl-induced Ca^2+^ signal and tension from T1 (red traces) to T2 (blue traces) in the presence of both BayK 8644 and TEA. ***B*,** Traces corresponding to Ca^2+^ signals measured in five ROIs in A (top traces) and force (bottom traces) measured in T1 (red trace) and T2 (blue trace) ends of the artery. The period of acquisition indicated by grey bar. ***C***, left and middle panels (***a*** and ***b***) showing average amplitude of KCl-evoked Ca^2+^ signal and force recorded at T1 and T2, respectively in the presence of BayK 8644 alone (*n=*7) and following addition of 10 mM TEA (*n=*7, expressed as % of peak KCl); ***C***, right panel (***c***) shows an average speed of KCl-evoked Ca^2+^ wave and constriction propagation measured between T1 (0 μm) and T2 (3000 μm) (*n=*7). The local Ca^2+^ signal and propagating Ca^2+^ wave induced by 1 s KCl pulse in the presence of BayK 8644 alone and following addition of TEA, respectively, can be seen in *Supplement Movie 5.* In this movie, the artery responded with a burst of three Ca^2+^ waves, two of each were initiated at the T1 and the third at the T2 (For interpretation of the references to colour in the text, the reader is referred to the web version of this article).

Ca^2+^ and vasoconstriction was measured at both downstream and upstream ends of denuded arteries (T1 and T2; See Methods for the details), with Ca^2+^ change measured in five ROIs: 0 μm, 500 μm, 1000 μm, 1500 μm, and 3000 μm (Fig. 5A*a*).

*BayK 8644 alone did not enable propagating responses to KCl.* In the presence of 1 μM BayK 8644, a 1 s KCl pulse raised local [Ca^2+^]_i_ by 18.6 ± 2.1% and vasoconstriction by 7.4 ± 0.4% (*n=*7) (Fig. 5Ab, B and C) only in the stimulated area (∼200–300 μm, Fig. 5B, top panel, green trace, Supplement Movie 5). The subsequent addition of 10 mM TEA led to the appearance of propagating intercellular Ca^2+^ waves and vasoconstriction (Fig. 5A*c*–*f*, Supplement Movie 5). Ca^2+^ waves propagated at 2.8 ± 0.2 mm s^−1^ (*n=*7, Fig. 5C*c*) from T1 to T2 with constant amplitude (Fig. 5B, top panel) and vasoconstriction at 2.7 ± 0.2 mm s^−1^ (*n=*7, Fig. 5C*c*). The amplitude of the Ca^2+^ wave and phasic contraction in the presence of TEA and BayK 8644 at T2 (51.2 ± 4.8, and 30.7 ± 1.2%) was not different from T1 (49.5 ± 3.4% and 28.3 ± 1.0%, *n=*7, Fig. 5C*ab*).

*TEA alone did not enable propagating responses to KCl.* In the presence of 10 mM TEA, 1 s KCl pulses only increased [Ca^2+^]_i_ at T1 (within ∼200-300 μm) to 15.4 ± 1.3% and vasoconstriction of 3.6 ± 1.1% (*n=*5). The subsequent addition of 1 μM Bay K 8644, as above, led to constant amplitude spreading responses at 2.8 ± 0.1 mm s^−1^ and 2.8 ± 0.2 mm s^−1^, for [Ca^2+^]_i_ and vasoconstriction from T1 to T2 (*n=*6), respectively. Thus, the ability of arteries to spread intercellular Ca^2+^ waves and phasic contractions was independent of the endothelium.

### 3.5 Spontaneous propagating intercellular Ca^2+^ waves and force in the presence of TEA and BayK 8644 in mesenteric arteries with intact endothelium

**Fig. 6:**
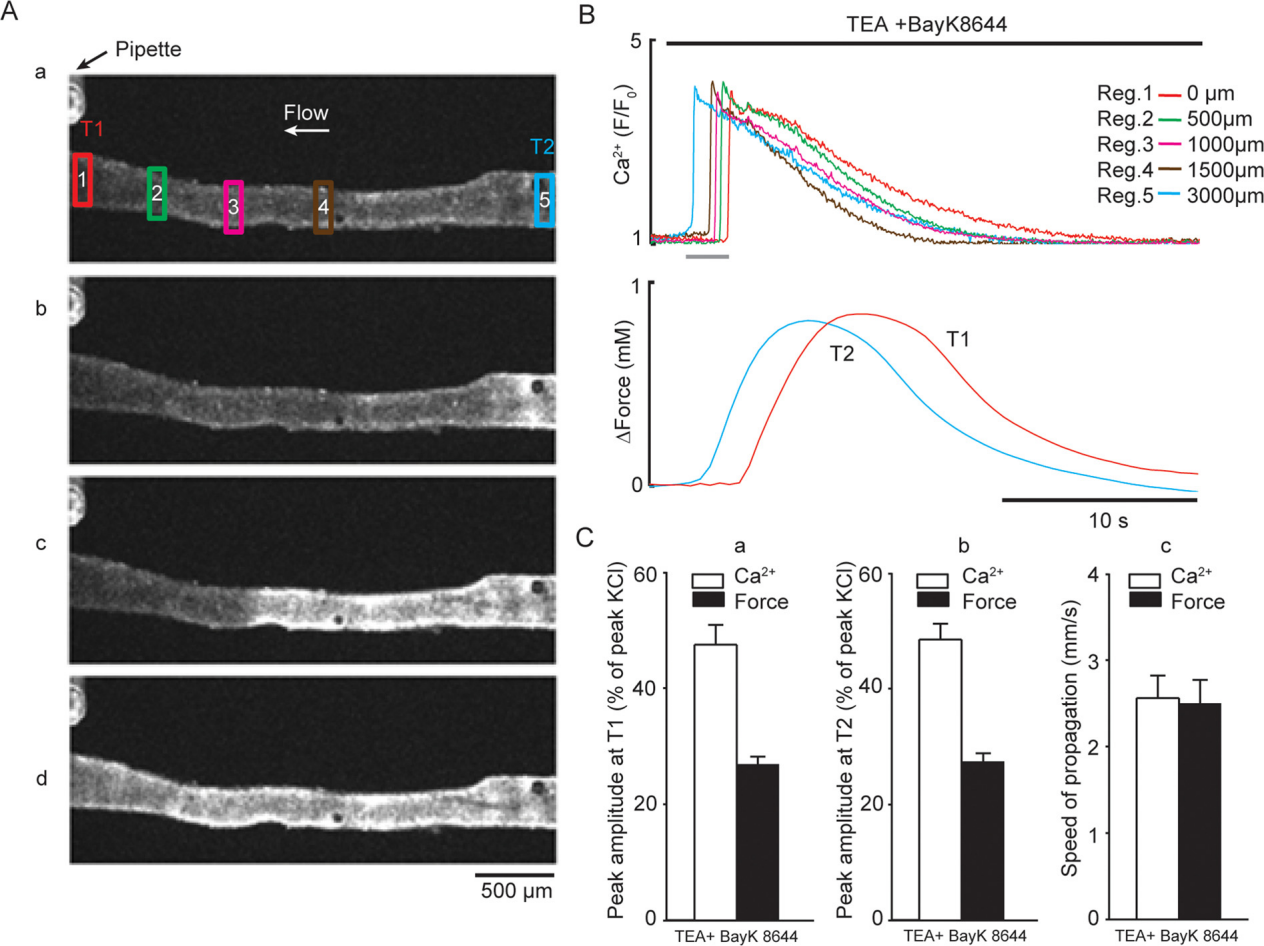
Spontaneous intercellular Ca^2+^ and mechanical waves induced by TEA in the presence of BayK 8644 in mesenteric arteries with intact endothelial layer. ***A***, Fluorescence images of mesenteric artery loaded with Ca^2+^ indicator, recorded at rest (***a***) and following incubation with 1 μM BayK 8644 and the addition of 10 mM TEA (***b-d***) indicated by bar at *B*. The interval between displayed images A(b–d) is 600 ms. Note rapid propagation of Ca^2+^ and mechanical waves from T2 (blue traces) to T1 (red traces). ***B***, Traces corresponding to Ca^2+^ transients (top traces) measured in five ROIs in *A* and force (bottom traces) recorded in T2 (blue trace) and T1 (red trace) ends of the artery. The period of acquisition indicated by grey bar. ***C***, left and middle panels (***a*** and ***b***) showing average amplitude of propagating single Ca^2+^ wave (*n=*5) and force (*n=*5, expressed as % of peak KCl) measured at T1 and T2, respectively; *C*, right panel (***c***) average speed of spontaneous Ca^2+^ and mechanical waves propagation measured between T1 (0 μm) and T2 (3000 μm) (*n=*5). The repetitive propagating Ca^2+^ waves described in *A–C* can be seen in *Supplement Movie 6* (For interpretation of the references to colour in the text, the reader is referred to the web version of this article).

In the presence of 10 mM TEA and 1 μM BayK 8644, over half the arteries studied developed spontaneous intercellular Ca^2+^ waves and vasoconstriction, which started at any point along the artery (Fig. 6, Supplement Movie 5 & 6). Ca^2+^ spread at 2.6 ± 0.3 mm s^−1^ with constant amplitude (Fig. 6A*b*–*d* and B top panel) and was accompanied by phasic vasoconstriction spreading at 2.5 ± 0.3 mm s^-1^ at both ends of the artery (*n=*5, Fig. 6B–C*ab*). Spontaneous intercellular Ca^2+^ waves and vasoconstriction in the presence of 10 mM TEA and 1 μM BayK 8644 were observed in denuded arteries as well.

### 3.6 Gap junction block prevented spontaneous propagation of Ca^2+^ and vasoconstriction in denuded arteries

**Fig. 7:**
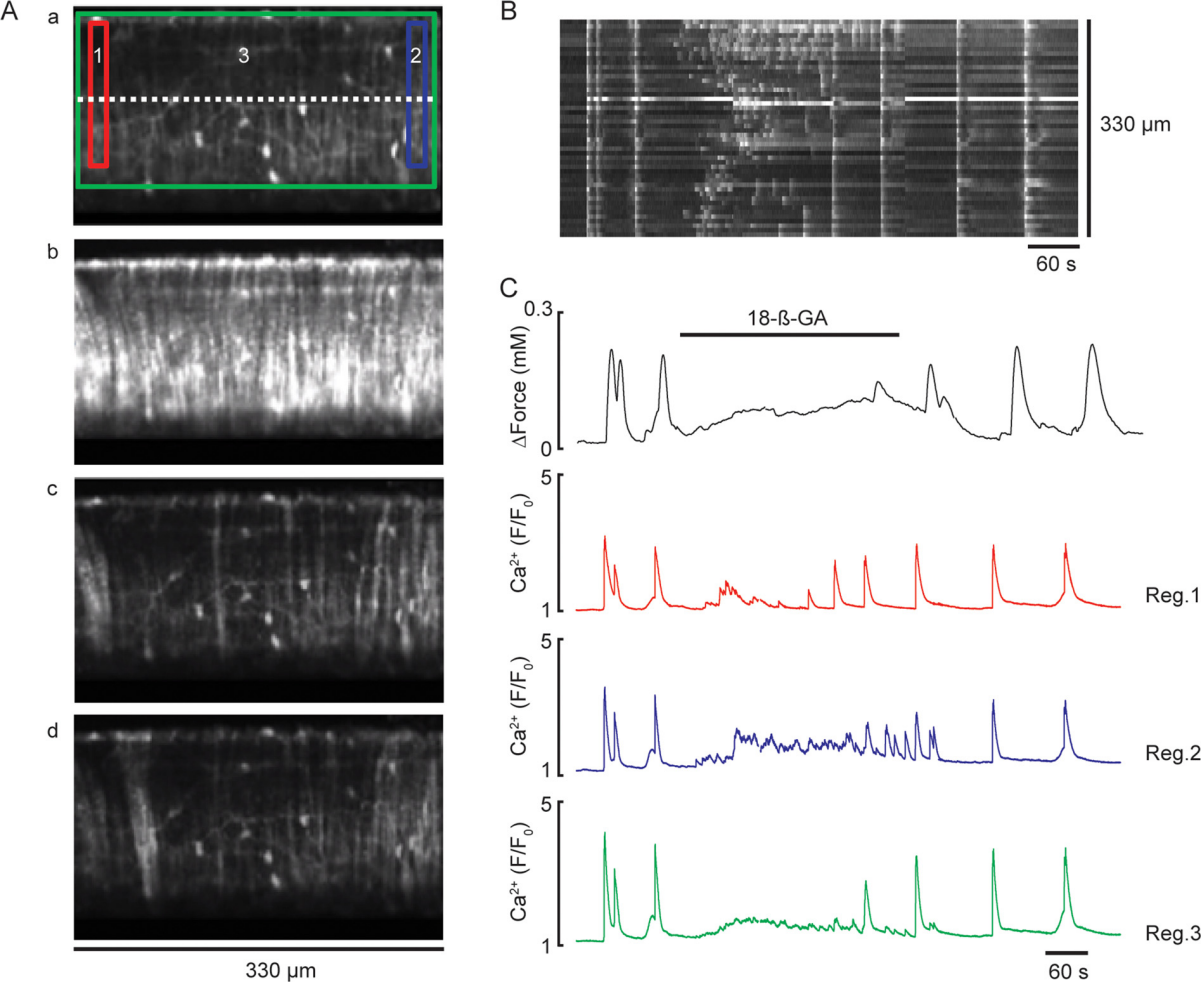
Effect of gap junction uncoupler, 18β-GA on spontaneous propagating intercellular Ca^2+^ waves and force of mesenteric artery loaded with Ca^2+^ indicator, in the presence of 10 mM TEA and 1 μM BayK 8644. ***A***, Fluorescent images showing MA at rest (***a***), during fully propagated intercellular Ca^2+^ wave (***b***), and at different time points in the presence of 20 μM 18β-GA (***c-d***). ***B***, Line-scan plot with respect to time, from SMCs of the whole segment of MA (dashed line indicated in *A*) showing extremely chaotic asynchronous spontaneous activity in individual SMCs. ***C***, Representative graph of 8 experiments showing changes in force (top panel) and Ca^2+^ signals recorded in three different ROIs (bottom panels) shown in Aa. Note, ROIs 1 (red) and 2 (blue) show the average Ca^2+^ signal in a small group of cells, while ROI 3 (green) shows the average Ca^2+^ signal acquired from the whole area of observation. Spontaneous Ca^2+^ waves and force described in A–C can be seen in *Supplement Movie 7* (For interpretation of the references to colour in the text, the reader is referred to the web version of this article).

The gap junction uncoupler 18β-GA (20 μM) did not prevent the appearance of spontaneous propagating intercellular Ca^2+^ waves and vasoconstriction in denuded arteries exposed to TEA and BayK 8644, but prevented spread (*n=*8, Fig. 7A*cd*, Supplement Movie 7), so synchronous Ca^2+^ waves now appeared randomly with associated vasoconstriction (Fig. 7A*cd*, B and C, Supplement Movie 7).

## 4 Discussion

### 4.1 Propagating intercellular Ca^2+^waves and vasoconstriction induced by TEA and BayK 8644

Arterial SMCs are generally quiescent, requiring agonist stimulation to evoke depolarization and vasoconstriction. In the presence of TEA (5–10 mM) arterial SMCs generate spike-like APs [[14], [15], [16], [17], [18]] sensitive to L-type VGCC blockers [17,18]. However, it is unclear whether these Ca^2+^ - based APs could propagate and as a result generate regenerative intercellular Ca^2+^ waves in vascular SMCs, in a similar manner to visceral smooth muscle. In the latter, Ca^2+^ influx via L-type VGCCs causes spike-like APs, which then give rise to propagating intercellular Ca^2+^ waves, which effectively synchronises contraction in a large group of SMCs [19,20]. In the current study, we show for the first time that in the presence of the K^+^ channels blocker TEA and L-type VGCC agonist BayK 8644, the propagating SMC Ca^2+^ transients accompanied by spreading vasoconstriction appears to be mediated by the conduction of APs through gap junctions. The generation and propagation of arterial spike-like APs is facilitated by the regenerative nature of L-type VGCCs.

BayK 8644 affects a 10-fold augmentation in inward Ca^2+^ current in myocytes isolated from rat MA [39]. We show that BayK 8644 can induce SMC APs in MAs, providing membrane TEA-sensitive K^+^ channels are blocked with TEA (5–10 mM). The APs establish propagating spikes of Ca^2+^ associated with spreading vasoconstriction, which can be blocked by the presence of the selective L-type Ca^2+^ channel blocker, nifedipine. Thus, L-type VGCC are essential for coupling between APs and intercellular Ca^2+^ waves, which then cause propagating vasoconstriction. In over half the arteries studied, propagating Ca^2+^ and mechanical waves occurred spontaneously in the presence of TEA and BayK 8644, and in all cases could be evoked by a brief (1 s) local application of 60 mM KCl. In each case, intercellular Ca^2+^ wave could propagate in a regenerative manner from the point of initiation, at a speed of around 3 mm s^−1^ and with constant amplitude (around 50% of peak Ca^2+^). The intercellular spread of Ca^2+^ appeared as a Ca^2+^ spike and was accompanied by vasoconstriction, also of constant amplitude (about 30% of peak force) and at a similar speed. Interestingly, neither was altered in the absence of the endothelium, these parameters were not significantly different. Indeed, in denuded arteries intercellular Ca^2+^ wave could propagate in a regenerative manner at similar speed 2.8 + 0.1 mm s^−1^ accompanied by vasoconstriction spreading at 2.8 + 0.2 mm s^−1^. This contrasts with skeletal muscle feed arteries, in which the spread of vasoconstriction initiated by depolarization to KCl was abolished when the endothelium was removed [11]. The rapid spread of Ca^2+^ could occur in either direction across the entire length of MA segments, and suggests gap junctions allow the transmission of AP, by cell-to-cell communication and without rectification. This required an active regenerative mechanism, as the Ca^2+^ signal caused by depolarization that did not initiate an AP decayed rapidly with distance. The regenerative propagating AP mediated by L-type VGCCs could be evoked provided K^+^ channels were blocked. Similar findings were observed in visceral SM e.g. guinea pig urinary bladder [19] and rat uterus [20]. The speed of Ca^2+^ wave propagation in guinea pig urinary bladder was around 1.6 mm s^−1^ [19], comparable to that found in MA in the present study (2.6 ± 0.3 mm s^−1^).

### 4.2 Role of homocellular gap junctions in control of propagating Ca^2+^ waves

In the current work, 18β-GA disrupted the propagation of intercellular synchronous Ca^2+^ transients, indicating that propagation was entirely due to the spread of APs via gap junctions. In visceral smooth muscle, the propagation of AP-mediated Ca^2+^ waves were also disrupted by gap junction blockers (e.g. [19]). In intact arterial segments regular propagating intercellular Ca^2+^ waves and synchronized contraction were inhibited in the presence of 18β-GA, but asynchronous Ca^2+^ spikes of limited spatial spread still appeared randomly within the arteriolar SMCs, indicating a continuing ability to initiate but not spread APs. Electrical coupling between SMCs enables depolarizing electrical signals to spread along the vessel wall and coordinate myogenic responses. These data are in a good agreement with our previous data, showing that myocytes and pericytes of ureteric microvessels *in situ* are electrically coupled and able to generate propagating intercellular Ca^2+^ waves across an arteriolar - venular network [40]. Our data also correlate well with the mechanical studies, indicating that TEA –induced spontaneous oscillations in tone in different arteries and arterioles are controlled by the smooth muscle layer, and independent of endothelium [41,42]. Barlett and colleagues [6] also suggested that vasoconstriction could be conducted in SMC layer, independently of endothelium, but in response to the α_1_-agonist phenylephrine. Both homocellular [43] and myoendothelial [33,44] gap junctions are present in SMCs in many vascular beds. Resistance and conduit arteries SMCs express predominantly Cx43 and Cx45 [[21], [22], [23], [24], [25],^30^,^31^], although a limited number of observations also indicate the presence of Cx37 and Cx40 in the SMCs [21,22,26,29]. However, in mesenteric arteries Cx37, Cx43 and Cx45 are found in homocellular gap junctions in the SMC layer [23,27,28,32], and Gustafsson and colleagues [45] detected Cx37, Cx40 and Cx43 plaques in the endothelium of mesenteric resistance arteries but failed to detect connexins in the medial cells. The reason for these apparent discrepancies is not clear but may reflect differences in methodology (1), heterogeneity in connexin expression between vascular beds (2), and branch order of vessel studied (3). Alternatively, they may suggest that cell-to-cell coupling via gap junctions in the media of resistance arteries is dynamic and subject to variability as a result.

In conclusion, while increasing the pulse duration of KCl enhanced the spread of depolarization and associated vasoconstriction, the spread was limited to less than 1500 μm. Block of current dissipation through K^+^ channels enabled the generation of regenerative Ca^2+^-based APs. These events could spread rapidly in either direction along the artery causing vasoconstriction. Homocellular gap-junctions between smooth muscle in the mesenteric artery enable AP spread without detriment and with no apparent detriment due to myoendothelial gap-junctions.

## Funding

This work was supported by the British Heart Foundation (BHF) (grant numbers PG/10/013/28221, FS/13/16/30199 and PG/14/58/30998).
