# Supplementary material for: Smooth muscle gap-junctions allow propagation of intercellular Ca2+ waves and vasoconstriction due to Ca2+ based action potentials in rat mesenteric resistance arteries
Source: Cell Calcium. 2018 Nov;75:21–9. doi: 10.1016/j.ceca.2018.08.001 (PMC6169741; doi:10.1016/j.ceca.2018.08.001)
Supplement: Supplementary file 1 [file mmc1.docx]

**Supporting information**

The following information is available in the online version of this article.

**Movie 1.** Contractile response of the downstream end of the artery (T1) evoked by local 10s 60mM KCl pulse. Corresponding to Figures 1*A* and 2*A*, this movie provides typical example of the local contraction of the downstream end of the artery seen as local deflection of the wires evoked by 10s 60mM KCl pulse.

**Movie 2.** Spatial spread of the fluorescently labelled beads and Ca^2+^ signal evoked by local 10s 60mM KCl pulse ejected from the T1 delivery tip. This movie shows spatial spread of fluorescently labelled beads corresponding to Figure 1*Ba* and Ca^2+^ signal evoked by 60mM KCl pulse corresponding to Figure 1*Bb.*

**Movie 3**. Spatial spread of Ca^2+^ signal evoked by local 10s 60mM KCl pulse ejected from the T1 delivery tip. Corresponding to Figure 2B*a-f* this movie shows spatial spread of the Ca^2+^ signal induced by 10s 60mM KCl pulse towards upstream direction T2. The traces in Figure 2*B* are derived from this recording.

**Movie 4.** Propagating intercellular Ca^2+^ wave in the MA arterial arcade with intact endothelium evoked by 1 s application of 60 mM KCl in the presence of TEA and BayK 8644. This movie shows that 1 s 60 mM KCl pulse in the presence of 1 µM BayK 8644 evokes local response, and in the presence of 1µMBayK 8644 and 10 mM TEA - propagating intercellular Ca^2+^ wave corresponding to Figure 4*Aa-e.* The traces in Fig. 4*B* are derived from this recording.

**Movie 5.** Propagating intercellular Ca^2+^ wave and force in the denuded arterial segment evoked by 1 s application of KCl in the presence of TEA and BayK 8644. This movie shows that 1 s 60 mM KCl pulse in the presence of 1 µM BayK 8644 evokes local Ca^2+^ transient corresponding to Fig. 5*Ab* and in the presence of 1 µM BayK 8644 and 10 mM TEA propagating intercellular Ca^2+^ wave corresponding to Fig. 5*Ac-f.* This recording shows an example when 1 s KCl pulse induced 2 consecutive Ca^2+^ waves propagating from the downstream T1 to the upstream end of the artery T2, followed by single Ca^2+^ wave initiated in the upstream end T2 and propagating to the downstream end T1 of the artery.

**Movie 6.** Spontaneous propagating intercellular Ca^2+^ waves observed in the presence of TEA and BayK 8644 in arteries with intact endothelium. According to Fig. 6*Aa-d* this movie shows a group of spontaneous intercellular Ca^2+^ waves initiated at T2 and propagating to the T1 end of the artery. The traces in Fig. 6*B* are derived from this recording.

**Movie 7.** Desynchronizing effect of gap junction blocker 18β-GA on arterial spontaneous propagating intercellular Ca^2+^ waves in denuded arteries. According to Fig. 7*Aa-d* this movie shows that gap junction blocker 18β-GA caused reversible inhibition of spontaneous fully propagating intercellular Ca^2+^ waves in MA treated with 1 µM BayK 8644 and 10 mM TEA. The line scan plot in Fig. 7*B* and traces in Fig. 7C are derived from this recording.
